# Atypical Spitz Nevi: A Case Report and Review of the Literature

**Published:** 2015-09-16

**Authors:** Amanda Filiberto, Christine Fuller, Jennifer Rhodes

**Affiliations:** ^a^Division of Plastic and Reconstructive Surgery; ^b^Department of Pathology, Virginia Commonwealth University School of Medicine, Richmond

**Keywords:** atypical Spitz nevi, pagetoid, malignant melanoma, histomorphology, comparative genomic hybridization

## Abstract

A case of atypical (“pagetoid”) compound Spitz nevus on the face of a 2-year-old girl is reported with a review of the literature. The nevus was composed of broad but laterally demarcated compound proliferation of enlarged fusiform and epithelioid melanocytes, with florid pagetoid scatter above the junction. Immunohistochemical analyses revealed the Ki-67 proliferation index to be relatively low. Given the histomorphological overlap with melanoma, an array-based comparative genomic hybridization approach revealed a subthreshold gain in chromosome 1q and gain in distal chromosome 17q, with no other associated chromosomal gains or losses. These molecular aberrations suggested a partially transformed tumor, without adequate evidence for a molecular diagnosis of melanoma. Because of the diagnostic dilemma posed by these lesions, recent research has focused on molecular alterations that may help differentiate Spitz tumors from malignant melanomas.

Spitz nevus is an uncommon melanocytic lesion composed of epithelioid and spindled cells, with a remarkable histological resemblance to malignant melanoma. First described in 1948, the diagnosis and management of Spitz tumors are still heavily debated today. Spitz nevi display a morphological and biological spectrum ranging from benign to malignant, with 3 main classifications: Spitz tumor, atypical Spitz tumor, and malignant melanoma.^1^ Atypical Spitz tumors, defined as a Spitz tumor with 1 or more atypical features, are a heterogeneous group with indeterminate biological potential. A wide spectrum of histopathological morphologies, classified as atypical Spitz nevi, has been reported and includes desmoplastic, angiomatoid, verrucous, plexiform, pagetoid, halo, myxoid, granulomatous, and tubular presentations.^2^ Even among expert dermatopathologists, there exists marked interobserver variability in diagnoses of atypical Spitz tumors and malignant melanoma. Because of the diagnostic dilemma posed by these lesions, recent research has focused on molecular alterations that may help differentiate Spitz tumors from malignant melanomas.

We present a case of 2-year-old girl with an atypical Spitz tumor, with peculiar clinical, histopathological, and molecular based findings.

## CASE REPORT

An otherwise healthy 2-year-old Caucasian female patient presented with a pigmented lesion on her left cheek since birth. The mother reported that the lesion had demonstrated recent enlargement. Medical and surgical history and systematic review were unremarkable. She had no family history of melanoma. Skin examination was notable for a 1-cm diameter heavily pigmented lesion with regular borders located at the junction on the lower eyelid. The rest of the skin examination was unremarkable.

Excisional biopsy with 1-mm margins was performed. Histopathological examination revealed a broad but laterally demarcated compound proliferation of enlarged fusiform and epithelioid melanocytes. Cells were distributed within a broad zone of acanthosis and relatively florid pagetoid scatter above the junction. Numerous well-formed pink globules were noted within the expanded epidermis, and eosinophilic hyalinization of dermal papillae were noted. Within the underlying dermis, there were maturing nests and cords of cytologically similar melanocytes coupled with an infiltrate of lymphocytes and melanophages. Rare mitotic figures were observed within the dermal component. Immunohistochemical analyses revealed the Ki-67 proliferation index to be relatively low.

Given the histomorphological overlap with melanoma, an array-based comparative genomic hybridization (CGH) approach was used on tumoral DNA extracted from the tissue block following microdissection.^3^ Results revealed a subthreshold gain in chromosome 1q and gain in distal chromosome 17q, with no other associated chromosomal gains or losses. These molecular aberrations suggested a partially transformed tumor, without adequate evidence for a molecular diagnosis of melanoma.

## DISCUSSION

Atypical Spitz nevi are described as conventional Spitz nevi with 1 or more atypical features with indeterminate biological potential. Features of atypical Spitz include larger size (>6 mm), irregular borders, irregular topography, or ulceration. On histology, asymmetry, ulceration, pagetoid melanocytosis, lack of zonation and maturation, deep mitoses, and atypia can be seen. There are no clear histomorphological criteria that differentiate atypical Spitz nevi from malignant melanoma.

A recent study among a panel of 13 expert dermatopathologists assessing atypical Spitz tumors found low interobserver agreement in categorizing lesions as malignant versus nonmalignant.^4^ Among 75 atypical Spitz tumor cases, there were 11 cases of malignant melanoma as evidenced by histomorphological appearance and disease progression. Although the majority of experts favored a diagnosis of melanoma in 7 of 11 cases (67%), there were 4 cases (36%) in which the majority of experts did not favor a diagnosis of melanoma. Notably, of the 2 cases with distant metastasis, only 1 or 2 of the 13 experts favored a diagnosis of melanoma. This highlights the ambiguity that still exists in the classification of atypical Spitz tumors. The misdiagnosis of a potentially malignant metastatic lesion as a benign atypical Spitz nevus by expert dermatopathologists is concerning, and further research into ancillary testing that can help elucidate clinical prognosis and classification of these lesions is warranted.

Ancillary studies including immunohistochemistry, CGH, and fluorescence in situ hybridization have proved promising to help distinguish melanocytic from nonmelanocytic lesions, but sensitivity and specificity of studies are not ideal. Immunohistochemical staining may be useful for detection of mitoses to determine the proliferation activity of a lesion. The rate of staining with proliferation markers is generally lower in atypical Spitz nevi than that in malignant melanomas. Increased expression of Ki-67, HMB-45, cyclin D1, and fatty acid synthase are more suggestive of melanoma.^5^ Both CGH and fluorescence in situ hybridization are methods that can determine DNA copy number changes associated with certain types of melanocytic lesions. Most atypical Spitz nevi do not reveal genomic aberrations, in contrast with melanomas, where more than 95% have been shown to have multiple chromosomal aberrations.^5^ However, borderline lesions often show borderline features at the cytogenetic level, and prognostic indication of these tests has yet to be clearly defined. For example, the array-based CGH results of this case showed gains in chromosome 17q and 1q, which suggest a partially transformed tumor without further molecular evidence in support of melanoma. As there are few reported cases of atypical Spitz nevi with chromosomal aberrations, the biological and prognostic significance of these findings are indeterminate at this time. Although there is still an inability to recommend objective guidelines for the optimal treatment of patients with atypical Spitz nevi,^6^ complete excision with clear margins and careful follow-up is highly recommended.

## CONCLUSION

This case report underlines the ambiguity and difficulty in diagnosing atypical Spitz nevi, and the clinical management is still a matter of debate. In support of the literature, it may be beneficial to view atypical Spitz nevi as a heterogeneous group of biologically different lesions rather than as a continuum. For example, the lesions can represent a large proportion of morphologically atypical but biologically benign lesions as well as a small group of true melanomas.^7^ It is important for dermatologists and plastic surgeons to be aware of atypical Spitz nevi, including pagetoid Spitz nevi, to avoid misdiagnosis and subsequent over- or undertreatment. Rigorous application of the microscopic diagnostic criteria is necessary to aid in the assessment of these lesions. In pediatric patients with atypical Spitz tumors, complete excision with greater clearance and careful follow-up is the management currently recommended.

## Figures and Tables

**Figure 1:**
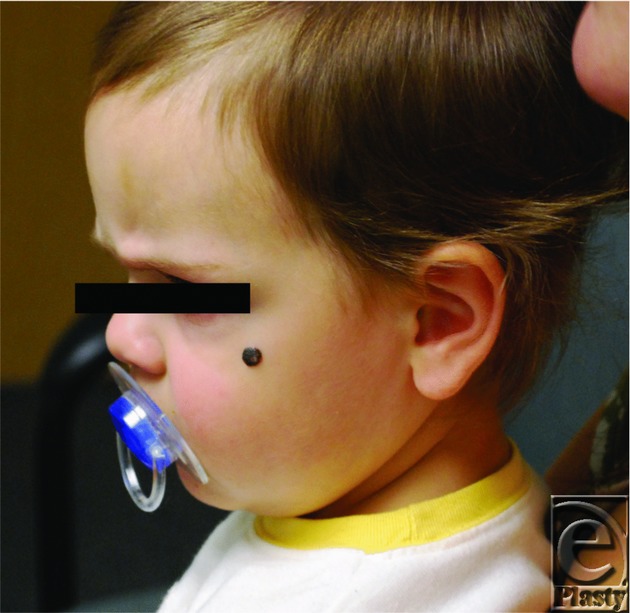
Nevus upon initial presentation to clinic. An otherwise healthy 2-year-old Caucasian female patient presented with a pigmented lesion on her left cheek since birth.

**Figure 2:**
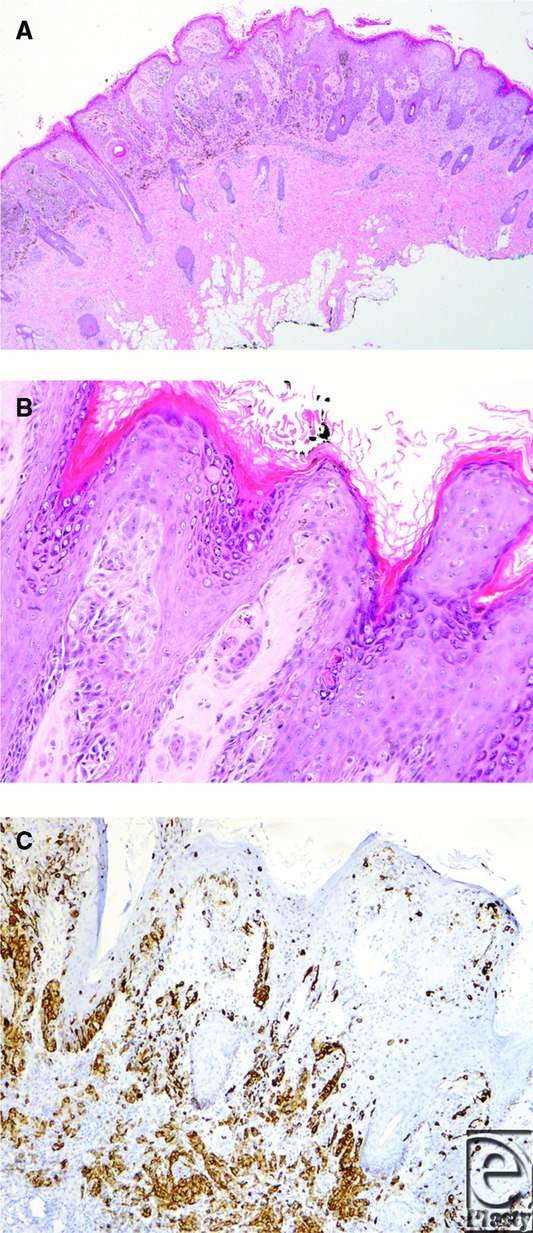
Photomicrographs of an atypical pagetoid Spitz nevus from a 2-year-old child. (a) At low power, melanocytic proliferation involves the epidermis and the underlying dermis, showing evidence of maturation toward the base of the lesion (H&sE, ×20). (b) Nests of epithelioid and fusiform melanocytes are noted at higher power, with obvious pagetoid single-cell extension above the junction (H&E, ×100). (c) Immunohistochemical stain targeting HMB-45 highlights the extent of pagetoid intraepidermal extension (anti-HMB-45, ×40). H&E indicates hematoxylin and eosin.

## References

[1] Barnhill RL (2006). The Spitzoid lesion: rethinking Spitz tumors, atypical variants, “Spitzoid melanoma” and risk assessment. Mod Pathol.

[2] Moscarella E, Al Jalbout S, Piana S (2015). The stars within the melanocytic garden: unusual variants of Spitz nevi. Br J Dermatol.

[3] McCalmont TH, Vemula S, Sands P, Bastian BC (2011). Molecular-microscopical correlation in dermatopathology. J Cutan Pathol.

[4] Gerami P, Busam K, Cochran A (2014). Histomorphologic assessment and interobserver diagnostic reproducibility of atypical Spitzoid melanocytic neoplasms with long-term follow-up. Am J Surg Pathol.

[5] Miteva M, Lazova R (2010). Spitz nevus and atypical Spitzoid neoplasm. Semin Cutan Med Surg.

[6] Massi D, Tomasini C, Senetta R (2015). Atypical Spitz tumors in patients younger than 18 years. J Am Acad Dermatol.

[7] Lallas A, Kyrgidis A, Ferrara G (2014). Atypical Spitz tumours and sentinel lymph node biopsy: a systematic review. Lancet Oncol.

